# Retroelements versus APOBEC3 family members: No great escape from the magnificent seven

**DOI:** 10.3389/fmicb.2012.00275

**Published:** 2012-08-14

**Authors:** Juan F. Arias, Takayoshi Koyama, Masanobu Kinomoto, Kenzo Tokunaga

**Affiliations:** Department of Pathology, National Institute of Infectious DiseasesTokyo, Japan

**Keywords:** retroelements, retrotransposition, LINE-1, Alu, APOBEC3, HIV-1, Vif, restriction factors

## Abstract

Retroelements comprise a large and successful family of transposable genetic elements that, through intensive infiltration, have shaped the genomes of humans and other mammals over millions of years. In fact, retrotransposons now account for approximately 45% of the human genome. Because of their genomic mobility called retrotransposition, some retroelements can cause genetic diseases; such retrotransposition events occur not only in germ cells but also in somatic cells, posing a threat to genomic stability throughout all cellular populations. In response, mammals have developed intrinsic immunity mechanisms that provide resistance against the deleterious effects of retrotransposition. Among these, seven members of the APOBEC3 (A3) family of cytidine deaminases serve as highly active, intrinsic, antiretroviral host factors. Certain A3 proteins effectively counteract infections of retroviruses such as HIV-1, as well as those of other virus families, while also blocking the transposition of retroelements. Based on their preferential expression in the germ cells, in which retrotransposons may be active, it is likely that A3 proteins were acquired through mammalian evolution primarily to inhibit retrotransposition and thereby maintain genomic stability in these cells. This review summarizes the recent advances in our understanding of the interplay between the retroelements currently active in the human genome and the anti-retroelement A3 proteins.

## Introduction

The evolution of vertebrate genomes has been driven in part by the long history of their interaction with genetic transposable elements. These so-called retrotransposons, which replicate via RNA intermediates, can be divided into two groups depending on the presence or absence of long terminal repeats (LTRs). LTR retrotransposons are endogenous retroviruses that constitute nearly 10% of murine and human genomes, but they have been rendered mostly inactive due to the accumulation of mutations, although some murine intracisternal A-particles (IAP) and MusD sequences remain viable (Dewannieux et al., 2004; Ribet et al., 2004). Non-LTR retrotransposons comprise the majority of transposable elements; in fact, collectively, they account for more than one third of the human genome. They can be further subdivided into three types; long interspersed elements (LINEs), short interspersed elements (SINEs), and the composite hominid-specific retrotransposons, each of which contain the only transposable elements currently active in the human genome, i.e., LINE-1, Alu, and *SINE-VNTR-Alu* (SVA), respectively (Deininger and Batzer, 2002; Ostertag et al., 2003).

Retrotransposition, discussed in greater detail below, involves the reverse transcription of an RNA intermediate with subsequent genomic integration in a process driven by retrotransposon-encoded RNA-dependent DNA polymerase and endonuclease. The integration of these elements may have harmful consequences for the host, compromising genomic stability via insertions, deletions, and DNA rearrangements and thereby posing a threat to human health, as described in several reports of retrotransposition-induced genetic disorders (Kazazian et al., 1988; Wallace et al., 1991; Kobayashi et al., 1998). In response, eukaryotic organisms have evolved mechanisms to restrict uncontrolled retrotransposition. Anti-retroelement strategies include transcriptional silencing through DNA methylation (Walsh et al., 1998; Bourc'his and Bestor, 2004; Burden et al., 2005), post-transcriptional silencing via RNA interference (Soifer et al., 2005; Yang and Kazazian, 2006), and some cellular factors inhibiting retrotransposition at the post-translational level. Of these cellular factors, seven members of the apolipoprotein B mRNA-editing catalytic polypeptide-like 3 (APOBEC3; referred to hereafter as the A3) family of cytidine deaminases have been shown to act as potent inhibitors of a wide range of both exogenous retroviruses and endogenous retroelements (Sheehy et al., 2002; Esnault et al., 2005; Chen et al., 2006; Kinomoto et al., 2007). In this review, we focus on active endogenous retroelements, their deleterious effects on the human genome, and the anti-retroelement activity of A3 proteins.

## Retrotransposons: an overview

**Figure 1:**
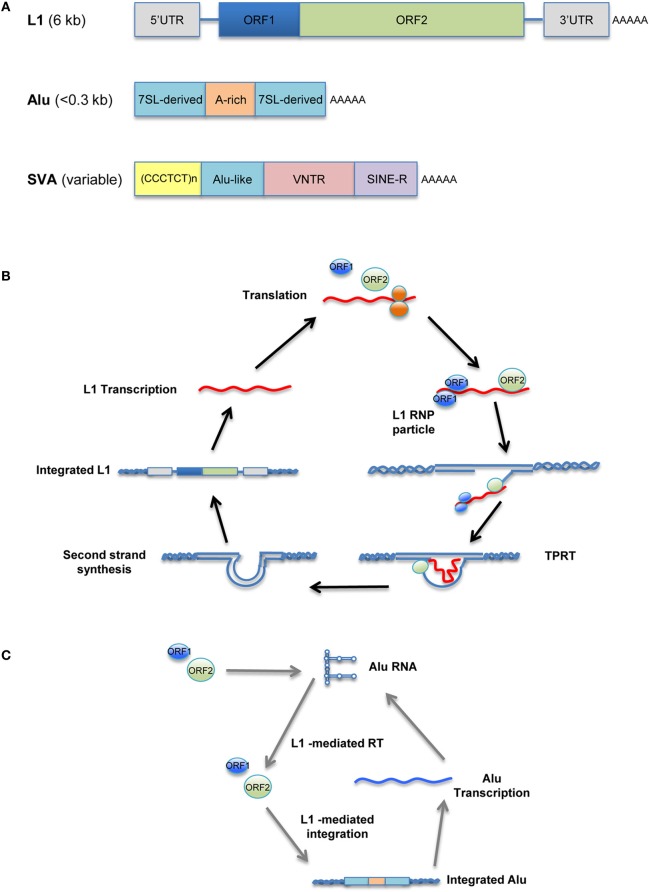
**Retrotransposition cycle.** Schematic representation of active human retrotransposons. **(A)** *Top*: L1 genomic organization, from the left: 5′ UTR, untranslated region; ORF-1, encoding an RNA-binding protein; linker region; ORF-2, encoding reverse transcriptase and endonuclease; 3′ UTR; AAA, poly(A) tail. *Middle*: Alu organization, from the left: 7SL-derived monomer; A-rich linker, A_5_TACA_6_; 7SL-derived monomer; AAA, poly(A) tail. *Bottom*: SVA organization, from the left: (CCCTCT)n, hexamer repeat; inverted Alu-like sequence; VNTR, variable number of tandem repeats; SINE-R, HERV-K-derived sequence; AAA, poly(A) tail. **(B)** Retrotransposition cycle: L1 elements are transcribed by RNA-polymerase II from an L1 promoter sequence. The L1 mRNA template is exported to the cytoplasm and translated. Retrotransposon-encoded proteins actively bind the L1 RNA transcript, forming a ribonucleoprotein particle (RNP) that is imported back into the nucleus. There, the L1-encoded endonuclease nicks an L1 target sequence (5′-TTTT/AA-3′) and the 3′-OH generated is used as a primer for target-primed reverse transcription (TPRT) by the L1-encoded reverse transcriptase, resulting in *de novo* integration into the host genome. **(C)** Alu as well as SVA elements are transcribed and hijack the L1-encoded enzymatic machinery to complete their respective retrotransposition cycles.

Unlike the murine LTR retrotransposons IAP and MusD, human versions, such as human endogenous retroviruses (HERV), have been mostly fossilized, and even those that are not are non-transposable. In contrast, many copies of human non-LTR retrotransposons can replicate through an RNA/protein complex intermediate and integrate into the host genome at a new site. The LINE retrotransposons, typified by LINE-1 (L1), account for approximately 17% of the human genome, corresponding to >500,000 copies (of which 100 copies are retrotransposition-competent). L1 retrotransposons are 6 kb in length and contain a 5′ untranslated region (UTR) that harbors a Pol II promoter; two ORFs necessary for their own replication; and a 3′ UTR containing a polyadenylation signal, followed by a poly(A) tail (Figure 1A, top). Briefly, L1 elements are first transcribed by RNA-polymerase II using a promoter located at the L1 5′ region (Ostertag and Kazazian, 2001). ORF1, encoding an RNA-binding protein, and ORF2, encoding a protein with reverse transcriptase and endonuclease activity, are then translated in the cytoplasm. The resulting proteins associate with L1 RNA to form a ribonucleoprotein (RNP) complex (Martin, 1991; Hohjoh and Singer, 1996; Figure 1B) that is transported back into the nucleus, where L1 is integrated into the host genome through a target-primed reverse transcription (Cost et al., 2002).

The human genome also contains more than 1 million copies of Alu elements; these are the most common SINE retrotransposons, representing 11% of our genome. The typical Alu element is approximately 300 bp in length and is formed by the fusion of two 7SL-RNA gene-derived monomers separated by an A-rich linker, followed by a poly(A) tail (Kriegs et al., 2007; Figure 1A, middle). Likewise, there are ~2700 copies of the composite SVA elements in the human genome. SVAs, which are approximately 2 kb long, are composed of CCCTCT hexameric repeats that are followed by an inverted Alu-like region, a region containing a variable number of tandem repeats (VNTRs), and a partial HERV-K *env*–LTR sequence termed SINE-R that ends with a polyadenylation signal, followed by a poly(A) tail (Ostertag et al., 2003; Figure 1A, bottom). Unlike L1, Alu and SVA elements are non-autonomous since they do not encode functional reverse transcriptase or endonuclease; instead, they use the enzymatic machinery of L1 for retrotransposition. Once Alu and SVA elements have been transcribed and exported to the cytoplasm, they hijack the L1-encoded enzymes in the vicinity of the ribosomes through mechanisms that are as-yet unclear (Figure 1C; Dewannieux et al., 2003; Ostertag et al., 2003).

## Retrotransposons in human diseases

Approximately 100 examples of disease-causing retrotransposon insertions are currently reported in the literature. It is estimated that *de novo* insertions of L1, Alu, and SVA elements are responsible for approximately 0.3% of all disease-causing human mutations, corresponding to event rates of 1:100, 1:20, and 1:900 births, respectively (Cordaux and Batzer, 2009). L1-induced genetic diseases include the following: Duchenne muscular dystrophy and X-linked dilated cardiomyopathy, resulting from insertions in the dystrophin gene (Narita et al., 1993; Yoshida et al., 1998); progressive chorioretinal degeneration, caused by the CHM gene disruption (van Den Hurk et al., 2003); hemophilia A and B, due to insertions in the factor VIII and IX genes, respectively (Kazazian et al., 1988; Li et al., 2001; Mukherjee et al., 2004); and chronic granulomatous disease, the result of a mutation arising from an insertion in the CYBB gene (Meischl et al., 2000). Genetic diseases linked to Alu integration events include neurofibromatosis via an insertion in the NF1 gene (Wallace et al., 1991; Wimmer et al., 2011); Apert syndrome, a severe autosomal dominant disorder, due to integration of the element into the fibroblast growth-factor receptor 2 (FGFR2) gene (Oldridge et al., 1999); and progressive renal failure (Dent's disease) due to disruption of the renal chloride channel (CLCN5) gene (Claverie-Martin et al., 2005). The involvement of SVA retrotransposition in human diseases has also been documented; namely, an insertion in the ARH gene leads to autosomal recessive hypercholesterolemia (Wilund et al., 2002); disruption of the BTK gene causes X-linked agammaglobulinemia (XLA; Rohrer et al., 1999); and disruption of the fukutin gene results in Fukuyama-type congenital muscular dystrophy (Kobayashi et al., 1998). Importantly, ongoing retrotransposon insertions seem to occur not only in germ cells and early embryos but also in brain tissues (Coufal et al., 2009; Baillie et al., 2011), somatic cells *in vitro* (Kubo et al., 2006; Rangwala et al., 2009), and somatic malignant tissues (Economou-Pachnis and Tsichlis, 1985; Morse et al., 1988; Miki et al., 1992). Several reports have also shown retrotransposon-induced recombination in certain types of cancer (Schichman et al., 1994; Jeffs et al., 1998).

## Cellular mechanisms limiting the activity of retroelements and retroviruses

As noted above, since unrestricted retrotransposition would result in genome instability, eukaryotic organisms have developed several strategies to restrict these mobile elements. Firstly, retrotransposition can be regulated at the transcriptional level through several transcription factors. For example, L1 transcription is positively regulated by SOX11 (Tchenio et al., 2000), RUNX3 (Yang et al., 2003) and YY1 (Athanikar et al., 2004), and negatively regulated by SRY (Tchenio et al., 2000) and SOX2 (Muotri et al., 2005). DNA methylation by the methyl-CpG-binding protein MeCP2 results in the repression of L1 transcription in neurons (Walsh et al., 1998; Burden et al., 2005; Muotri et al., 2010). Secondly, retrotransposable elements are also susceptible to post-transcriptional regulation. For instance, endogenously encoded small interfering RNAs have been shown to reduce L1 retrotransposition *in vitro* (Soifer et al., 2005; Yang and Kazazian, 2006). Additionally, L1 transcripts that contain multiple polyadenylation signals lead to premature polyadenylation, resulting in the attenuation of L1 activity via truncation of its full-length transcripts (Perepelitsa-Belancio and Deininger, 2003). Thirdly, some cellular factors regulate retrotransposition at the post-translational level. In mice, the 3′–5′ exonuclease Trex1 digests retroelement-derived DNA to suppress the autoimmune response (Stetson et al., 2008), Consistent with this, mutations in human Trex1 cause autoimmune diseases like familial chilblain lupus and Aicardi-Goutieres syndrome (Crow et al., 2006). Likewise, HIV-1 restriction factors such as the cytidine deaminases, the focus of this review, can inhibit L1 and Alu retrotransposition through a mechanism that is still unknown.

**Figure 2:**
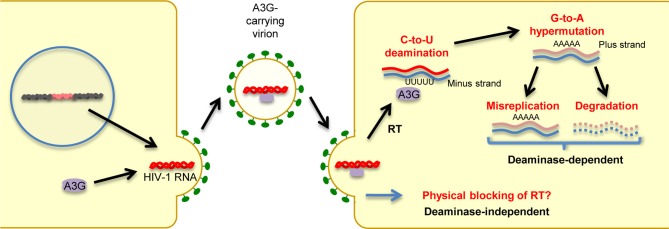
**Mechanisms of antiretroviral and anti-retroelement activity of A3G protein.** A3G potently restricts Vif-deficient HIV-1 viruses: A3G is incorporated into virus particles in virus-producing cells. Following the infection of target cells, A3G inhibits viral replication either by binding to HIV-1 RNA, leading to physical blocking of reverse transcription (deaminase-independent mechanism), or by deaminating the viral minus-strand DNA during reverse transcription, thus generating G → A hypermutations in the proviral DNA plus strand.

In humans, the cellular cytidine deaminase family comprises several members, including activation-induced cytidine deaminase (AID), APOBEC1, APOBEC2, the A3 family, and APOBEC4 (Harris and Liddament, 2004; Conticello, 2008; Smith et al., 2012). APOBEC1 is the catalytic subunit of an RNA-editing complex that deaminates C^6666^→U in the mRNA of the lipid-transport protein apolipoprotein B, thereby creating a premature stop codon that leads to a truncated protein in gastrointestinal tissues (Teng et al., 1993). APOBEC1 proteins from multiple small-animal species exhibit inhibitory activity against not only exogenous retroviruses (Ikeda et al., 2008) but also endogenous retroviruses, such as murine IAP and MusD sequences, as well as L1 elements (Ikeda et al., 2011). AID plays a role in the adaptive humoral immune system by inducing somatic hypermutations and class switch recombination, which allows affinity maturation and memory development; however, its precise mechanism of action remains to be determined (Honjo et al., 2005). As described in detail in a subsequent section, members of the A3 family are potent inhibitors of both exogenous retroviruses and endogenous retroelements. A3G, the most extensively studied member of the A3 family, was the first cytidine deaminase shown to restrict infection by Vif-deficient HIV-1 viruses. Briefly, as depicted in Figure 2, A3G is incorporated into budding virions and thus exerts its antiviral effect at the post-entry step in target cells, either by mediating extensive deamination of the minus-strand of viral DNA during reverse transcription, which results in G → A hypermutations in the proviral DNA plus strand (deaminase-dependent mechanism) (Harris et al., 2003; Mangeat et al., 2003; Zhang et al., 2003), or by binding to HIV-1 RNA, leading to physical impairment of reverse transcription (deaminase-independent mechanism; Newman et al., 2005; Bishop et al., 2006; Iwatani et al., 2007). Consequently, primate lentiviruses have evolved to counteract the antiretroviral activity of A3G by acquiring Vif. This accessory protein prevents A3G incorporation into virions through its proteasomal degradation (Marin et al., 2003; Sheehy et al., 2003; Stopak et al., 2003). We and others have shown that Vif proteins derived from different HIV-1 subtypes differ in their potency of A3G inhibition, suggesting differential levels of viral fitness among clades (Iwabu et al., 2010; Binka et al., 2012). APOBEC2, a cardiac- and skeletal muscle-specific cytidine deaminase, is required for muscle development and early embryogenesis (Etard et al., 2010; Sato et al., 2010; Vonica et al., 2011). The physiological role of APOBEC4 remains to be determined.

## Differential antiviral and anti-retroelement activities of A3 cytidine deaminases

Members of the A3 family contain either single (A3A, A3C, A3H) or double (A3B, A3DE, A3F, and A3G) cytidine deaminase domains (CDA). In A3G and A3F, the N-terminal CDA is responsible for RNA-dependent oligomerization, while the C-terminal CDA mainly mediates the deamination of single-stranded DNA (Hache et al., 2005; Newman et al., 2005). Some A3 family members strongly inhibit a wide range of exogenous retroviruses, as well as other viral pathogens, including herpesviruses, parvoviruses, papillomaviruses, and hepadnaviruses (Baumert et al., 2007; Vartanian et al., 2008; Narvaiza et al., 2009; Suspène et al., 2011b). The importance of A3 proteins *in vivo* has been demonstrated in murine studies in which mice lacking the A3 gene were shown to be more susceptible to viral infection than their wild-type counterparts (Okeoma et al., 2007, 2009; Takeda et al., 2008). A3 proteins also inhibit the mobilization of endogenous retroviruses, such as MusD, IAP, and the yeast LTR-retrotransposon Ty1 (Esnault et al., 2005; Schumacher et al., 2008), in addition to their inhibitory activity on L1 and Alu retrotransposition. The gene copy number of A3 family members is species-specific in mammals, in which except for primates, one, two, or three A3 proteins are encoded, whereas in humans and in non-human primates, seven A3 proteins have been recognized (A3A, A3B, A3C, A3DE, A3F, A3G, and A3H; Sawyer et al., 2004; OhAinle et al., 2006). Of note, expansion of the A3 gene cluster in primate genomes correlates with a sharp reduction in retrotransposition activity, suggesting that these restriction factors have evolved to protect mammalian hosts from retroelements (Sawyer et al., 2004; Schumann, 2007). Antiretroviral and anti-retroelement potencies were shown to differ in the seven members of A3 family, independently of their subcellular localization (Kinomoto et al., 2007). However, the exact mechanism by which A3 proteins inhibit retrotransposition is unclear. The current findings on antiviral and anti-retroelement activities of A3 members are summarized below and in Table 1.

**Table 1:** **Antiviral and anti-retroelement spectrum of A3 family members**.

**A3 family**	**Exogenous**		**Endogenous**	
	**Non-retroviruses**	**Retroviruses**	**Non-human retroelements**	**Human retroelements**
**A3A** (C/N)	AAV (Chen et al., 2006; Narvaiza et al., 2009)	HIV-1 (Peng et al., 2007; Koning et al., 2011; Schmitt et al., 2011)	IAP (Chen et al., 2006; Ikeda et al., 2011) MusD (Bogerd et al., 2006a; Chen et al., 2006)	L1 (Bogerd et al., 2006b; Chen et al., 2006; Muckenfuss et al., 2006; Kinomoto et al., 2007; Niewiadomska et al., 2007; Tan et al., 2009; Khatua et al., 2010; Ikeda et al., 2011)
	HPV (Vartanian et al., 2008)	SIV (Schmitt et al., 2011)	PERV (Dörrschuck et al., 2011)	Alu (Bogerd et al., 2006b; Muckenfuss et al., 2006; Niewiadomska et al., 2007; Tan et al., 2009; Khatua et al., 2010)
		HTLV-1 (Ooms et al., 2012)		Reconstituted HERV-K (Lee et al., 2008)
**A3B** (N)		HIV-1 (Bishop et al., 2004; Doehle et al., 2005a; Bogerd et al., 2006a; Kinomoto et al., 2007; Hultquist et al., 2011) SIV (Yu et al., 2004)	IAP (Bogerd et al., 2006a; Chen et al., 2006; Ikeda et al., 2011) MusD (Chen et al., 2006; Ikeda et al., 2011)	L1 (Bogerd et al., 2006b; Muckenfuss et al., 2006; Stenglein and Harris, 2006; Kinomoto et al., 2007; Niewiadomska et al., 2007; Khatua et al., 2010; Ikeda et al., 2011; Wissing et al., 2011)
		HTLV-1 (Ooms et al., 2012)	PERV (Dörrschuck et al., 2011)	Alu (Bogerd et al., 2006b; Muckenfuss et al., 2006; Stenglein and Harris, 2006; Niewiadomska et al., 2007)
		MLV (Doehle et al., 2005b; Kinomoto et al., 2007)		Reconstituted HERV-K (Lee et al., 2008)
		RSV (Wiegand and Cullen, 2007)		
**A3C** (C/N)	HBV (Baumert et al., 2007; Köck and Blum, 2008) HPV (Vartanian et al., 2008)	HIV-1 (Bishop et al., 2004; Yu et al., 2004; Bogerd et al., 2006a; Hultquist et al., 2011)	IAP (Chen et al., 2006) MusD (Chen et al., 2006)	L1 (Bogerd et al., 2006b; Chen et al., 2006; Muckenfuss et al., 2006; Kinomoto et al., 2007; Niewiadomska et al., 2007; Khatua et al., 2010)
	HSV-1 (Suspène et al., 2011b)	SIV (Yu et al., 2004) PFV (Russell et al., 2005; Perković et al., 2009)	Ty1 (Dutko et al., 2005)	Alu (Bogerd et al., 2006b; Muckenfuss et al., 2006; Niewiadomska et al., 2007; Khatua et al., 2010)
	EBV (Suspène et al., 2011b)	MLV (Langlois et al., 2005; Kinomoto et al., 2007)		
**A3DE** (C)		HIV-1 (Dang et al., 2006; Hultquist et al., 2011)		L1 (Stenglein and Harris, 2006; Kinomoto et al., 2007; Niewiadomska et al., 2007; Duggal et al., 2011)
		SIV (Dang et al., 2006)		Alu (Tan et al., 2009)
**A3F** (C)		HIV-1 (Wiegand et al., 2004; Zheng et al., 2004; Bishop et al., 2006; Holmes et al., 2007; Yang et al., 2007)	IAP (Chen et al., 2006) MusD (Chen et al., 2006)	L1 (Turelli et al., 2004a; Muckenfuss et al., 2006; Stenglein and Harris, 2006; Kinomoto et al., 2007; Niewiadomska et al., 2007; Khatua et al., 2010)
		SIV (Bogerd et al., 2004; Mangeat et al., 2004; Schröfelbauer et al., 2004)	Ty1 (Dutko et al., 2005; Schumacher et al., 2005)	Reconstituted HERV-K (Lee and Bieniasz, 2007; Lee et al., 2008)
		XMRV (Paprotka et al., 2010)	PERV (Dörrschuck et al., 2011)	
		PFV (Russell et al., 2005; Delebecque et al., 2006)		
		MLV (Langlois et al., 2005)		
		RSV (Wiegand and Cullen, 2007)		
		MPMV (Doehle et al., 2006)		
**A3G** (C)	HBV (Turelli et al., 2004a; Köck and Blum, 2008)	HIV-1 (Harris et al., 2003; Mangeat et al., 2003; Zhang et al., 2003; Newman et al., 2005; Bishop et al., 2006)	IAP (Esnault et al., 2005; Ikeda et al., 2011)	L1 (Kinomoto et al., 2007; Niewiadomska et al., 2007; Khatua et al., 2010; Ikeda et al., 2011)
		SIV (Bogerd et al., 2004; Mangeat et al., 2004; Schröfelbauer et al., 2004)	MusD (Esnault et al., 2005; Chen et al., 2006; Schumacher et al., 2008; Ikeda et al., 2011)	Alu (Chiu et al., 2006; Hulme et al., 2007; Bulliard et al., 2009; Tan et al., 2009)
		MLV (Harris et al., 2003; Kobayashi et al., 2004; Doehle et al., 2005b; Langlois et al., 2005; Kinomoto et al., 2007)	Ty1 (Dutko et al., 2005; Schumacher et al., 2005)	Reconstituted HERV-K (Lee et al., 2008)
		XMRV (Groom et al., 2010;, Paprotka et al., 2010)	PERV (Jónsson et al., 2007; Dörrschuck et al., 2011)	
		PFV (Russell et al., 2005; Delebecque et al., 2006)		
		MMTV (Okeoma et al., 2007)		
		EIAV (Bogerd et al., 2008)		
		HTLV-1 (Sasada et al., 2005; Fan et al., 2010)		
		RSV (Wiegand and Cullen, 2007)		
		MPMV (Doehle et al., 2006)		
**A3H** (C/N)	HPV (Vartanian et al., 2008)	HIV-1 (OhAinle et al., 2008; Harari et al., 2009; Li et al., 2010; Zhen et al., 2010; Wang et al., 2011)	PERV (Dörrschuck et al., 2011)	L1 (Kinomoto et al., 2007; OhAinle et al., 2008; Tan et al., 2009)
	HBV (Köck and Blum, 2008)	HTLV-1 (Ooms et al., 2012)		Alu (Tan et al., 2009)

(C), cytoplasmic localization; (N), nuclear localization; (C/N), diffuse cytoplasmic/nuclear localization; AAV, Adeno-associated virus; HPV, Human papillomavirus; HBV, Hepatitis B virus; HSV-1, Herpes simplex virus 1; EBV, Epstein-Barr virus; MLV, Murine leukemia virus; RSV, Rous sarcoma virus; PFV, Primate foamy virus; XMRV, Xenotropic murine leukemia virus-related virus; MPMV, Mason-Pfizer Monkey Virus; MMTV, Mouse mammary tumor virus; HTLV-1, Human T-cell leukemia virus type 1; EIAV, Equine infectious anemia virus; IAP, Intracisternal A particles; PERV, Porcine endogenous retrovirus; L1, Long interspersed element 1; HERV-K, Human endogenous retrovirus K.

## A3A

Human A3A (hA3A) lacks inhibitory activity against HIV-1 produced from 293T cells overexpressing this protein (Bishop et al., 2004; Bogerd et al., 2006a; Kinomoto et al., 2007; Hultquist et al., 2011), since it is not incorporated into virions (Goila-Gaur et al., 2007; Aguiar et al., 2008). In human monocytic cells as targets, however, hA3A blocks the early phase of HIV-1 infection (Peng et al., 2007; Koning et al., 2011) but is counteracted by the HIV-2/SIV (simian immunodeficiency virus) accessory protein Vpx (Berger et al., 2010, 2011). Also, hA3A can inhibit infections by adeno-associated virus (Chen et al., 2006; Narvaiza et al., 2009), human papillomavirus (HPV; Vartanian et al., 2008), porcine endogenous retrovirus (PERV; Dörrschuck et al., 2011), and human T-cell leukemia virus type 1 (HTLV-1; Ooms et al., 2012). Importantly, *in vitro* overexpression experiments have demonstrated that hA3A effectively inhibits the retrotranspositions of L1, Alu (Bogerd et al., 2006b; Chen et al., 2006; Muckenfuss et al., 2006; Kinomoto et al., 2007; Niewiadomska et al., 2007; Tan et al., 2009; Khatua et al., 2010; Ikeda et al., 2011), IAP, and MusD (Bogerd et al., 2006a; Chen et al., 2006; Ikeda et al., 2011) through a deaminase-independent mechanism. hA3A is intrinsically able to restrict infection of the genetically reconstituted HERV-K in a deaminase-dependent manner (Lee et al., 2008). In a recent report, hA3A was shown to induce somatic hypermutation in human mitochondrial and nuclear DNA; in the latter, this included genes associated with the development of cancer (Suspène et al., 2011a).

## A3B

Human A3B (hA3B) is the sole member of the A3 family with an exclusive nuclear localization (Bogerd et al., 2006a; Muckenfuss et al., 2006; Stenglein and Harris, 2006; Kinomoto et al., 2007; Pak et al., 2011), sharing a common nuclear import mechanism with AID (Lackey et al., 2012). hA3B inhibits infections of HIV-1 and SIV, independently of the presence of Vif (Bishop et al., 2004; Yu et al., 2004; Doehle et al., 2005a; Hultquist et al., 2011). Since hA3B expression is extremely low in target CD4^+^ T-cells (Bishop et al., 2004; Doehle et al., 2005a; Koning et al., 2009; Refsland et al., 2010), Vif might have failed to evolve the mechanism to antagonize this antiretroviral factor. Also, hA3B restricts infections by murine leukemia virus (MLV; Doehle et al., 2005b; Kinomoto et al., 2007), PERV (Dörrschuck et al., 2011), HTLV-1 (Ooms et al., 2012), and Rous sarcoma virus (RSV; Wiegand and Cullen, 2007). Like hA3A, *in vitro* overexpression of hA3B inhibits the retrotranspositions of L1, Alu (Bogerd et al., 2006b; Muckenfuss et al., 2006; Stenglein and Harris, 2006; Kinomoto et al., 2007; Niewiadomska et al., 2007; Khatua et al., 2010; Ikeda et al., 2011), IAP, and MusD (Bogerd et al., 2006a; Chen et al., 2006; Ikeda et al., 2011) in a deaminase-independent manner, while inhibiting the reconstituted HERV-K infection through a deaminase-dependent mechanism (Lee et al., 2008). In a recent study, endogenously expressed hA3B effectively restricted L1 retrotransposition in both transformed cells and human embryonic stem cells (Wissing et al., 2011).

## A3C

Human A3C (hA3C) is abundantly expressed in numerous tissues and cell types (Jarmuz et al., 2002), and its expression is unresponsive to interferon-α (Koning et al., 2009). Although hA3C is efficiently incorporated into retroviral particles, it exhibits only partial antiviral activity against HIV-1, with or without Vif (Bishop et al., 2004; Yu et al., 2004; Bogerd et al., 2006a; Hultquist et al., 2011). By contrast, hA3C is able to efficiently block the replication of SIV, which also encapsidates this protein but is readily antagonized by SIV Vif (Yu et al., 2004). hA3C can inhibit infection of primate foamy virus (PFV), which carries an hA3 antagonistic Bet protein (Russell et al., 2005; Perković et al., 2009). The overexpression of hA3C results in a moderate inhibition of L1 and Alu retrotranspositions (Bogerd et al., 2006b; Chen et al., 2006; Muckenfuss et al., 2006; Kinomoto et al., 2007; Niewiadomska et al., 2007; Khatua et al., 2010) but effectively inhibits those of IAP, MusD, and Ty1 (Dutko et al., 2005; Chen et al., 2006). In a recent study, hA3C was shown to restrict infections by MLV (Langlois et al., 2005; Kinomoto et al., 2007), hepatitis B virus (HBV; Baumert et al., 2007; Köck and Blum, 2008), HPV (Vartanian et al., 2008), herpes simplex virus 1 and Epstein-Barr virus (Suspène et al., 2011b).

## A3DE

Human A3DE (hA3DE) overexpression has moderate effects on L1 and Alu retrotransposition (Stenglein and Harris, 2006; Kinomoto et al., 2007; Niewiadomska et al., 2007; Tan et al., 2009; Duggal et al., 2011). Similarly, hA3DE exhibits low levels of anti-HIV-1 and anti-SIV activities, both of which are antagonized by the respective Vif proteins (Dang et al., 2006; Hultquist et al., 2011). The reduced activity is determined by a cysteine residue located at amino acid position 320 of hA3DE. Substitution with the corresponding tyrosine present in A3F resulted in a 20-fold increase of A3DE activity (Dang et al., 2011). Indeed, the chimpanzee version of A3DE, carrying a tyrosine residue at this position, shows much higher antiretroviral activity, while both human and chimpanzee A3DEs exhibit similar levels of inhibition against retroelements, suggesting that the host defense activity of A3DE against retroelements has been evolutionarily conserved (Duggal et al., 2011).

## A3F/A3G

With regard to the antiretroviral potencies of human A3G (hA3G) and A3F (hA3F) proteins, overwhelming amount of information is well-summarized elsewhere (Harris and Liddament, 2004; Huthoff and Towers, 2008; Malim, 2009). Similar to hA3G, as introduced in the previous section, hA3F has been shown to potently restrict the replication of Vif-deficient HIV-1 viruses in target cells after its incorporation into budding virions through both deaminase-dependent and -independent mechanisms (Bishop et al., 2004; Wiegand et al., 2004; Zheng et al., 2004; Holmes et al., 2007; Yang et al., 2007). The *in vitro* overexpression of hA3G inhibits not only retroviral infections, such as those by HTLV-1 (Sasada et al., 2005; Fan et al., 2010), SIV (Bogerd et al., 2004; Mangeat et al., 2004; Schröfelbauer et al., 2004), PFV (Russell et al., 2005; Delebecque et al., 2006), equine infectious anemia virus (Bogerd et al., 2008), MLV (Harris et al., 2003; Kobayashi et al., 2004; Doehle et al., 2005b; Langlois et al., 2005; Kinomoto et al., 2007), Mason-Pfizer monkey virus (MPMV; Doehle et al., 2006), xenotropic murine leukemia virus-related virus (XMRV; Groom et al., 2010;, Paprotka et al., 2010), PERV (Jónsson et al., 2007; Dörrschuck et al., 2011), and RSV (Wiegand and Cullen, 2007), but also retrotranspositions of non-human LTR retroelements, such as IAP, MusD, and Ty1 (Dutko et al., 2005; Esnault et al., 2005; Chen et al., 2006; Schumacher et al., 2008; Ikeda et al., 2011). This cytidine deaminase is also effective against HBV (Turelli et al., 2004a; Köck and Blum, 2008). With regard to potential to inhibit L1 retrotransposition, conflicting results have been reported; some lines of evidence suggest that hA3G has anti-Alu activity (Chiu et al., 2006; Hulme et al., 2007; Bulliard et al., 2009; Tan et al., 2009) but little or no anti-L1 activity (Turelli et al., 2004b; Bogerd et al., 2006b; Muckenfuss et al., 2006; Stenglein and Harris, 2006). We and others, however, have shown that hA3G is also able to restrict L1 retrotransposition, albeit less potently than hA3A or hA3B, through deaminase-independent mechanisms (Kinomoto et al., 2007; Niewiadomska et al., 2007; Khatua et al., 2010; Ikeda et al., 2011). These discrepancies might be due to cell-type differences in hA3 protein expression levels, as we have described previously (Kinomoto et al., 2007). A putative mechanism of L1 inhibition by hA3G is as follows: When L1 forms the RNP complex in the cytoplasm (Figure 1B, right half), cytoplasmic hA3G protein might be able to access the complex through the interaction with L1 RNA, and then enter the nucleus together with the complex. This could result in the effective inhibition of L1 reverse transcription, by physically blocking the access to the chromosomal DNA, or by impeding the movement of the reverse transcriptase on a template L1 RNA. In the case of the infection by reconstituted HERV-K, hA3G carries out deamination showing only marginal inhibition of infectivity (Lee et al., 2008). Among the viruses and retroelements described above, hA3F is known to inhibit infections of PFV (Russell et al., 2005; Delebecque et al., 2006), MLV (Langlois et al., 2005), XMRV (Paprotka et al., 2010), MPMV (Doehle et al., 2006), PERV (Dörrschuck et al., 2011), RSV (Wiegand and Cullen, 2007), and reconstituted HERV-K (Lee and Bieniasz, 2007; Lee et al., 2008), as well as the retrotransposons; L1 (Chen et al., 2006; Muckenfuss et al., 2006; Stenglein and Harris, 2006; Kinomoto et al., 2007; Niewiadomska et al., 2007; Khatua et al., 2010), Ty1 (Dutko et al., 2005; Schumacher et al., 2005), MusD and IAP (Chen et al., 2006).

## A3H

Human A3H (hA3H) is the most distantly related of the hA3 members and is known for its functional polymorphisms. Currently, four major haplotypes (I–IV) have been identified in human populations, among which haplotype I has the highest allelic frequencies (OhAinle et al., 2008). Haplotypes I, III, and IV generate unstable proteins with very little, if any, antiretroviral and anti-retroelement activity. Haplotype II, however, expresses a stable protein with relatively high inhibitory activity on HIV-1 (OhAinle et al., 2008; Harari et al., 2009; Li et al., 2010; Zhen et al., 2010; Wang et al., 2011) and HTLV-1 (Ooms et al., 2012), and its overexpression effectively restricts L1 retrotransposition (OhAinle et al., 2008; Tan et al., 2009). These observations suggest that the relative lack of anti-retroviral and anti-retroelement potencies in hA3H is not due to insufficient enzymatic activity but to the instability of the protein. It should be noted that hA3H haplotype II is mainly localized to the cytoplasm, while the haplotype I protein passively diffuses into the nucleus (Li and Emerman, 2011). The ability of hA3H to block infections of HPV (Vartanian et al., 2008), HBV (Köck and Blum, 2008), and PERV (Dörrschuck et al., 2011) has also been reported, although the responsible haplotypes have not been described.

## Concluding remarks

Retrotransposable elements have successfully proliferated over tens of millions of years of mammalian evolution, such that they now constitute 45% of the human genome. Retrotransposition spreads DNA fragments to different genomic sites and is thus considered to be one of the driving forces in genome evolution by contributing to the formation of new genes. On the other hand, the price to pay for such genomic innovation, in which retrotransposons integrate in their host genomes, is the potential disruption of essential genes, resulting in deleterious effects, some of which are clearly associated with genetic diseases and tumorigenesis. Consequently, to prevent uncontrolled retrotransposition, host organisms have evolved several defense mechanisms. Among these, the seven members of A3 family have the ability to restrict not only a broad range of exogenous retroviruses but also endogenous retroelements, as described herein. Interestingly, high-level A3 expression is seen in the testis and ovary and in embryonic stem cells (Jarmuz et al., 2002; Bogerd et al., 2006b; OhAinle et al., 2006), in which the retroelements are hypomethylated and therefore active (Bourc'his and Bestor, 2004; Dupressoir and Heidmann, 1996). These findings support the evolutionary acquisition of A3 proteins to protect these cells primarily from the genomic instability caused by the disruptive effect of endogenous retroelements. Further investigations of A3-mediated intrinsic immunity are likely to provide insights into the molecular mechanisms of the host defenses that do not allow retrotransposons to escape from the seven members of A3.

### Conflict of interest statement

The authors declare that the research was conducted in the absence of any commercial or financial relationships that could be construed as a potential conflict of interest.
